# Key to Smith’s Diagram of Parliamentary Rules

**Published:** 1886-01

**Authors:** 


KEY TO SMITH'S DIAGRAM OF PARLIAMENTARY RULES. Together with concise hints and directions for conducting the business of deliberative assemblies. By Uriah Smith. Second Edition Revised. Battle Creek, Michigan, Review & Herald Publishing Association. 1885.

The subject in this little book is presented under an entirely new arrangement, by which a great amount of information is presented to the eye at once in a marvelously condensed form. By an ingeniously-devised system of diverging and converging lines, all the rules applying to any given motion, and all the motions coming under any given rule, are presented at one view, facilitating immensely the acquisition of a general knowledge of this subject, and furnishing to a chairman instant information on any point upon which doubts may arise. It was in use by the President of the Georgia Senate at the last meeting of that body.

